# Thromboembolic event rate in paroxysmal and persistent atrial fibrillation: Data from the GISSI-AF trial

**DOI:** 10.1186/1471-2261-13-28

**Published:** 2013-04-15

**Authors:** Marcello Disertori, Maria Grazia Franzosi, Simona Barlera, Franco Cosmi, Silvia Quintarelli, Chiara Favero, Glauco Cappellini, Gianna Fabbri, Aldo Pietro Maggioni, Lidia Staszewsky, Luigi Andrea Moroni, Roberto Latini

**Affiliations:** 1 Department of Cardiology, Santa Chiara Hospital, Trento, Italy; 2 Department of Cardiovascular Research, IRCCS - Istituto di Ricerche Farmacologiche Mario Negri, Milan, Italy; 3 Department of Cardiology, Valdichiana Santa Margherita Hospital, Cortona, (AR), Italy; 4 ANMCO Research Center, Florence, Italy; 5 Department of Cardiology, Villa Scassi Hospital, Genova, Italy; 6 GISSI-AF Coordinating Center, ANMCO Research Center, via La Marmora 34, Florence, 50121, Italy

**Keywords:** Paroxysmal atrial fibrillation, Persistent atrial fibrillation, Thromboembolic risk, Warfarin, Atrial fibrillation recurrences

## Abstract

**Background:**

Few data on the thromboembolic (TE) risk of paroxysmal and persistent atrial fibrillation (AF) are available. This study aimed to assess the incidence of TE events in paroxysmal and persistent AF.

**Methods:**

We performed a subset post hoc analysis of 771 patients with paroxysmal and 463 with persistent AF enrolled in the multicenter, prospective, randomized, double-blind, placebo-controlled GISSI-AF trial - comparing the efficacy of valsartan versus placebo in preventing AF recurrences – where the choice of antithrombotic treatment was left to the judgment of the referring physician. TE and major outcome events were centrally validated. AF recurrences were detected by frequent clinic visits and a transtelephonic monitoring device with weekly and symptomatic transmissions.

**Results:**

Eighty-five percent of patients had a history of hypertension, and the 7.7% had heart failure, left ventricular dysfunction, or both. The mean CHADS_2_ score was 1.41±0.84. TE and major bleeding events were observed at a low incidence among the overall population at 1-year follow-up (0.97% and 0.81%, respectively). The univariate and multivariable analyses revealed no statistically significant differences in the incidence of TE, major bleeding events or mortality in paroxysmal and persistent AF patients. TE events were more common among women than men (p=0.02). The follow-up examination showed under- or overtreatment with warfarin in many patients, according to guideline suggestions. Warfarin was more frequently prescribed to patients with persistent AF (p<0.0001) and patients with AF recurrences (p<0.0001). AF recurrences were noninvasively detected in 632 (51.2%) patients. In patients without AF recurrences, the TE event rate was 0.5% versus 1.74%, 1.28%, and 1.18% for those with only symptomatic, only asymptomatic or both symptomatic and asymptomatic AF recurrences, respectively, but the difference was not statistically significant, even after adjusting for warfarin treatment and the CHADS_2_ score (HR 2.93; CI 95%; 0.8-10.9; p=0.11).

**Conclusions:**

TE and major bleeding events showed a very low incidence in the GISSI-AF trial population, despite under- or overtreatment with warfarin in many patients. TE events had a similar rate in paroxysmal and persistent AF.

**Trial registration:**

Trial registration number: NCT00376272

## Background

Atrial fibrillation (AF) is the most common cardiac arrhythmia, and stroke is its most feared complication [1]. Warfarin treatment significantly reduces the risk of stroke [2]. The thromboembolic (TE) risk varies greatly in different clinical settings and in relation to the presence of several risk factors. Therefore, the benefit/risk ratio of warfarin is not favorable for all patients [3]. Several TE risk stratification schemes allow for the selection of a tailored therapeutic approach. However, these schemes are mostly validated in permanent AF patients [4-6].

The available data on TE risk in paroxysmal and persistent AF are limited. The TE risk for patients with paroxysmal AF is comparable with that of permanent AF patients in some but not all studies [7-11]. However, only one study [11] has provided information regarding the TE risk in patients with paroxysmal versus persistent AF, and a more extensive assessment is needed.

The present study assesses the incidence of TE events in paroxysmal versus persistent AF in the GISSI-AF patients (Gruppo Italiano per lo Studio della Sopravvivenza nell’Infarto Miocardico – Atrial Fibrillation) [12].

## Methods

The rationale, design, and results of the GISSI-AF trial have been published previously [12] (Clinical Trials.gov Identifier: http://NCT00376272). Briefly, the GISSI-AF was a prospective, multicenter, randomized, double-blind, placebo-controlled trial that assessed whether the addition of the angiotensin II receptor blocker valsartan to established therapies reduced the recurrence of AF in patients with a history of AF associated with cardiovascular diseases. All of the treatments that were prescribed for AF or underlying cardiovascular diseases were allowed, including antithrombotic therapy, with a strong recommendation to follow the available AF guidelines. Patients were eligible for randomization if they had experienced at least two electrocardiographically documented episodes of symptomatic AF in the previous six months or had undergone a successful cardioversion (electrical or pharmacological) between 14 days and 48 hours prior to randomization. Study visits were scheduled at 2, 4, 8, 24, and 52 weeks. Two weeks after randomization, all of the patients were provided with a transtelephonic monitoring device (Cardiobios 1, Telbios S.p.A., Italy), and they had to activate this tool when experiencing symptoms or at least once a week. From November 2004 to January 2007, 1,442 patients were randomized at 114 centers in Italy. The Steering Committee designed and supervised the GISSI-AF trial [12]. The Ethics Committees at all of the participating centers approved the study, and all of the patients signed an informed consent.

The primary objective of the present analysis was to assess the incidence of TE events in paroxysmal versus persistent AF. Secondary objectives were i) the incidence of major bleeding events, mortality and hospitalizations in paroxysmal versus persistent AF; ii) antithrombotic treatment at baseline and during the follow-up; and iii) the incidence of TE events in patients with and without AF recurrences. Ischemic stroke, transient ischemic attack (TIA), and systemic embolism were classified as TE events. Intracranial hemorrhage and bleeding requiring hospitalization or transfusion were classified as major bleeding events. The revised CHADS_2_ score [4] was used to define TE risk (e.g., heart failure, hypertension, age, diabetes, prior stroke or TIA double). A score of 0 represents low risk; 1 is intermediate and ≥2 is high. The OBRI score [13] was used to define the bleeding risk (e.g., age, prior stroke, prior gastrointestinal bleeding, one or more recent myocardial infarctions, hematocrit <30%, serum creatinine concentration >1.5 mg/dl, diabetes). A score of 0 represents a low risk, 1–2 represents an intermediate risk, and 3–4 represents a high risk.

### Type of AF

To be enrolled in the GISSI-AF study, patients were required to be in sinus rhythm (SR) for at least two days prior to randomization. We classified AF according to the guidelines established at the start of the study (ACC/AHA/ESC 2006) [1]. AF was defined as paroxysmal if the AF was self-terminating, usually within 48 hours, although AF could continue for up to 7 days; AF was defined as persistent when the AF episodes lasted longer than 7 days. Arrhythmia termination by cardioversion did not change the classification of AF. In the overall GISSI-AF population (1,442 patients), 771 patients were categorized as paroxysmal AF and 463 as persistent AF. In 208 patients, the duration of AF was uncertain, leading to difficulty in categorizing the arrhythmia [1,14], and these patients were excluded from the analysis.

### Statistical methods

The baseline characteristics of the patients grouped by AF duration (paroxysmal or persistent) were compared using the chi-squared or Fisher’s test for categorical variables and the t test for continuous variables. Patients with an uncertain AF duration were excluded from the analysis. The occurrence of TE events and major hemorrhagic events for paroxysmal and persistent AF patients during the study was represented by Kaplan-Meier curves and compared using the log rank test. The association between the type of AF (persistent versus paroxysmal) and study outcomes was assessed using a univariate Cox proportional hazards models. Cox multivariable models evaluated the independent association of the type of AF with the outcomes, adjusting for the baseline variables that were significantly related to the outcomes in the univariate analysis (p<0.05). The use of antithrombotic treatments at baseline and at the 6- and 12-month follow-up visits was determined according to the type of AF, CHADS_2_ score, and the presence of at least 1 AF recurrence using the chi-squared test. The association between AF recurrences (either symptomatic or asymptomatic) and TE events that occurred during the study was assessed using univariate and multivariable Cox models, after adjusting for warfarin treatment and CHADS_2_ score. All of the probability values are two-tailed. The statistical analyses were performed using SAS software, version 9.2 (SAS Institute, Cary, NC, USA).

## Results

### Patient characteristics

Among all of the patients enrolled in GISSI-AF (n=1442), 1,234 patients had either paroxysmal AF (n=771) or persistent AF (n=463). The baseline characteristics are shown in Table 1. Patients with paroxysmal AF were younger and included more females and had a higher number of previous AF episodes, higher hypertension incidence and more frequent use of class I antiarrhythmic agents. Patients with persistent AF more frequently had heart failure or left ventricular dysfunction and used angiotensin-converting enzyme inhibitors, aldosterone blockers or amiodarone. In paroxysmal AF, SR restoration was spontaneous in 134 and by cardioversion in 637 patients. Cardioversion was performed in the first 48 hours for 533 (69.13%) patients and between 48 hours to 7 days in 104 (13.48%) patients. No significant differences in the TE risk factors were observed between those with paroxysmal or persistent AF (CHADS_2_ score), but the bleeding risk (OBRI score) was lower for the paroxysmal AF patients.

**Table 1:** Baseline characteristics of patients with paroxysmal or persistent AF

**Baseline patient characteristics**	**Paroxysmal AF (n = 771)**	**Persistent AF (n = 463)**	**P value**
**Demographics**			
Age – years (mean±SD)	66.75±9.84	68.78±8.54	0.0002
Age classification:			
• <65 years old	298 (38.65)	133 (28.73)	0.0016
• 65-75 years old	304 (39.43)	219 (47.30)	
• ≥75 years old	169 (21.92)	111 (23.97)	
Female sex	351 (45.53)	136 (29.37)	<0.0001
**AF details**			
≥2 episodes of AF in the previous 6 months	433 (56.68)	110 (24.72)	<0.0001
Cardioversion	637 (82.62)	453 (97.84)	<0.0001
Time to cardioversion ≤48 hours	533 (69.13)	0	-
Time to cardioversion >48 hours - ≤7 days	104 (13.48)	0	-
Time to cardioversion >7 days - ≤3 months	0	260 (56.15)	-
Time to cardioversion >3 months	0	193 (41.68)	-
**Coexisting conditions**			
HF/Left ventricular ejection fraction <40%	32 (4.15)	63 (13.61)	<0.0001
Stroke/TIA/Systemic embolism	48 (6.23)	27 (5.83)	0.7790
Hypertension for 6 months or more	677 (87.81)	372 (80.35)	0.0004
Diabetes mellitus	115 (14.92)	64 (13.82)	0.5976
Documented coronary disease	34 (4.41)	19 (4.10)	0.7973
Peripheral artery disease	28 (3.63)	20 (4.32)	0.5450
Renal dysfunction	19 (2.46)	14 (3.02)	0.5553
Neoplasia	27 (3.5)	11 (2.38)	0.2676
Alcohol abuse	6 (0.78)	10 (2.16)	0.0378
**Cardiovascular therapies**			
• No antithrombotic treatment	182 (23.61)	17 (3.67)	<0.0001
• Warfarin	192 (24.90)	404 (87.26)	
• Antiplatelet agent	385 (49.94)	24 (5.18)	
• Warfarin + antiplatelet agent	12 (1.56)	18 (3.89)	
ACE inhibitors	411 (53.31)	275 (59.40)	0.0372
Valsartan	389 (50.45)	238 (51.40)	0.7466
Aldosterone blockers	17 (2.20)	55 (11.88)	<0.0001
Class I antiarrhythmic agents	304 (39.43)	113 (24.41)	<0.0001
Amiodarone	181 (23.48)	219 (47.30)	<0.0001
**Risk stratification schemes for thromboembolic events**			
CHADS_2_ - score			
• 0	55 (7.13)	38 (8.21)	0.3946
• 1	448 (58.11)	251 (54.21)	
• ≥2	268 (34.76)	174 (37.58)	
**Risk stratification schemes for bleeding events**			
OBRI - score			
• 0	248 (32.17)	113 (24.41)	0.0120
• 1-2	518 (67.19)	345 (74.51)	
• 3-4	5 (0.65)	5 (1.08)	

### Outcomes

**Figure 1:**
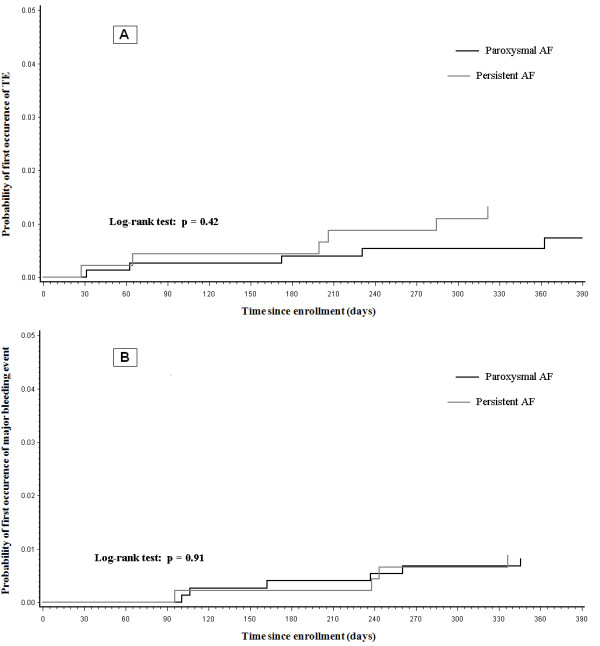
Kaplan Meier curves for TE (panel A) and major bleeding events (panel B) during the follow-up period in paroxysmal and persistent AF patients.

Twelve patients (0.97%) died during the 1-year follow-up period. TE and major bleeding events occurred in 12 (0.97%) and 10 (0.81%) patients, respectively (Table 2). TE events were more common in women (p=0.02). The univariate and multivariable analyses did not show significant differences for TE, major bleeding, death, cumulative TE/major bleeding/death events, or hospitalization rate for cardiovascular or any other reasons between the paroxysmal and persistent AF patients (Table 3). TE and major bleeding events were uniformly observed during the follow-up period for paroxysmal and persistent AF patients (Figure 1), while AF recurrences were particularly frequent during the first 2 months [12].

**Table 2:** **TE and major bleeding events according to the antithrombotic treatment, gender and CHADS**_**2 **_**score**

**Total (%)**		**Antithrombotic treatment at the time of event occurrence**		
		Warfarin*	**Antiplatelet**	**Untreated**
**Thromboembolic events**	12 (0.97)	5	6	1
Stroke	4	2	2	0
TIA	4	1	2	1
Systemic embolism	4	2	2	0
Female	9	4	5	0
Male	3	1	1	1
CHADS_2_ score				
0	1	1	0	0
1	4	2	1	1
≥ 2	7	2	5	0
**Major bleeding events**	10 (0.81)	5	5	0
Intracranial bleeding	5	3	2	0
Systemic bleeding	5	2	3	0
Female	7	5	2	0
Male	3	0	3	0
CHADS_2_ score				
0	1	1	0	0
1	4	1	3	0
≥ 2	5	3	2	0

*Includes patients taking both warfarin and antiplatelet treatment.

**Table 3:** Univariate and multivariable Cox regression analyses of persistent versus paroxysmal AF on patient outcomes at 1-year follow-up

	**Paroxysmal AF (n=771)**	**Persistent AF (n=463)**	**Unadjusted HR [95% CI] Persistent vs. Paroxysmal**	**p value**	**Adjusted HR [95% CI] Persistent vs. Paroxysmal**	**p value**
**TE events**	6 (0.78)	6 (1.30)	1.60 [0.51 – 4.95]	0.42	2.14 [0.68 – 6.79]	0.20
**Death**	9 (1.17)	3 (0.65)	0.54 [0.15 - 2.00]	0.36	0.52 [0.13 – 2.03]	0.35
**Major bleeding events**	6 (0.78)	4 (0.86)	1.08 [0.30 – 3.82]	0.90	0.78 [0.19 – 3.22]	0.73
**TE + death + major bleeding events**	20 (2.59)	11 (2.38)	0.89 [0.43 - 1.85]	0.75	0.84 [0.38 -1.85]	0.67
**Hospitalization for any reason**	148 (19.20)	97 (20.96)	1.07 [0.83 - 1.38]	0.60	0.94 [0.71 - 1.24]	0.65
**Hospitalization for a CV event**	123 (15.95)	78 (16.85)	1.03 [0.78 -1.37]	0.84	0.86 [0.64 - 1.17]	0.2681

### Antithrombotic therapy

**Figure 2:**
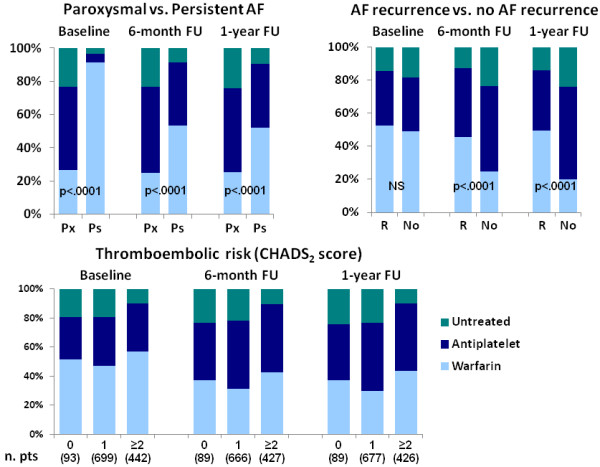
**The percentage of patients who used antithrombotic treatments at baseline and at the 6-month and 1-year follow-up (FU) examinations according to the type of AF (Px = paroxysmal AF; Ps = persistent AF), the presence of at least 1 AF recurrence (R) and the CHADS**_**2 **_**score.** The warfarin group also includes patients receiving both warfarin and an antiplatelet agent.

The use of antithrombotic treatments at baseline and at the 6- and 12-month follow-ups, according to the type of AF, CHADS_2_ score, and the presence of at least 1 AF recurrence, is reported in Figure 2.

Warfarin treatment, at baseline and during the follow-up, was significantly more common among patients with persistent versus paroxysmal AF (p<0.0001) and in patients with AF recurrences versus those without recurrences (p<0.0001). A reduction in warfarin use was observed during the follow-up. This reduction was independent of the TE risk level. For the high TE risk patients (CHADS_2_≥2), only 42.9% and 43.4% remained on warfarin treatment at the 6- and 12-month follow-up examinations, respectively (undertreatment). For the low TE risk patients (CHADS_2_=0), 37.1% remained on warfarin treatment at both the 6- and 12-month follow-up examinations (overtreatment). After excluding the patients with a cardioversion in the previous month, which may have led to the initiation of warfarin treatment, the net overtreatment at the 6-month follow-up was 35.2% [15]. The TE event rate was 1.57% among the undertreated patients versus 0.86% for those treated appropriately, while the major bleeding event rate was 2.08% among the overtreated patients versus 0.76% for those treated appropriately. However, these differences were not statistically significant.

### AF recurrences

During the 1-year follow-up, 1,839 AF recurrences occurred in 632 (51.2%) patients, of which 45.5% had only symptomatic recurrences, 24.7% had only asymptomatic recurrences, and 13.4% had both symptomatic and asymptomatic recurrences; for the remaining patients the information on symptoms was incomplete [16]. Symptomatic AF recurrences were more frequent in paroxysmal AF patients, while asymptomatic recurrences were more frequent in persistent AF patients (p<0.0001). In patients without AF recurrences, the TE event rate was 0.5% versus 1.74%, 1.28%, and 1.18% in patients with symptomatic, asymptomatic and both symptomatic and asymptomatic AF recurrences, respectively. Even after adjusting for warfarin treatment and the CHADS_2_ score, the difference was not statistically significant (HR 2.93; CI 95%; 0.8-10.9; p=0.11).

### Transtelephonic AF monitoring

Among the overall population, 45,575 transtelephonic transmissions of ECGs were received weekly, with a compliance rate of 77%, which was stable during the 1-year follow-up. AF was centrally recognized and validated for 836 cases (1.8%). In addition, 2,260 transtelephonic ECGs were transmitted from symptomatic patients, 752 of which (33.2%) were centrally validated as AF.

## Discussion

Our study shows a very low incidence of TE events in the GISSI-AF population despite under- or overtreatment with warfarin in many patients. We did not observe significant differences in TE or in other outcome events between patients with paroxysmal or persistent AF.

### The outcomes for paroxysmal and persistent AF

Scarce data on the TE risk for paroxysmal and persistent AF are available. In particular, few data on persistent AF exist because several previous trials analyzed persistent and permanent AF together [7-10]. Analyses of paroxysmal and persistent AF are further complicated because patients differ greatly in symptom frequency, duration and type. There are also differences in the classification of AF across studies.

A retrospective analysis of the SPAF trial [7] compared patients with intermittent AF to patients with permanent AF, all treated with antiplatelet agents, and reported a similar annualized incidence of TE events for both types of AF (3.2% versus 3.3%). In a subanalysis of the ACTIVE W trial [8], patients with persistent or permanent AF were grouped together as patients with sustained AF, showing that patients with paroxysmal AF had a risk for TE events comparable to patients with sustained AF (2.0% versus 2.25%, respectively). In the observational SCAF study [10], the incidence of TE events was similar for patients with paroxysmal AF (2.6%) or permanent AF (2.9%). In contrast, in the SPORTIF III and V trials [9], in which all of the patients were treated with warfarin or ximelagatran, the TE event rate was lower in patients with paroxysmal compared to permanent AF (0.93% and 1.73%, respectively). In the Euro Heart Survey of AF [11], the TE event rates for paroxysmal and persistent AF were reported to be 2.8% and 2.7%, respectively, compared to 5.1% of permanent AF patients at the 1-year follow-up; in this study, paroxysmal and persistent AF were categorized using the same criteria for the present analysis. Unfortunately, we did not have a control group of patients with permanent AF due to the study design of the GISSI-AF trial.

In the GISSI-AF trial, the TE event rate (0.97%) for the paroxysmal and persistent AF patients was unexpectedly low compared to previous studies. The rate was nearly half of the TE event rate reported by the ACTIVE W study [8] and nearly one-third of the TE event rate reported by the SPAF [7] and the SCAF [10] studies and by Euro Heart Survey [11] on AF. Several possible explanations exist for these results. 1) The lower incidence of TE events in our patients compared to previous trials on paroxysmal and persistent AF may be related to different TE risks at baseline. The mean CHADS_2_ score of the GISSI-AF patients (1.41±0.84) was lower than the ACTIVE W (1.79±1.03) and SCAF (1.7±1.3) patients, although the difference in CHADS_2_ scores does not seem to be large enough to justify the variable incidence of TE events. 2) The lower incidence of TE events could be due to inaccurate reporting; however, all of the clinical events in the GISSI-AF trial were regularly monitored and centrally adjudicated to ensure data quality. 3) The risk of TE events among AF patients has progressively decreased in recent years, perhaps reflecting a better control of hypertension and other risk factors [3,17]. 4) A reconsideration of the TE risk in paroxysmal and persistent AF based on new TE risk variables, together with the known clinical risk factors, may be required.

In the GISSI-AF trial, aside from non-significant differences in the TE event rate, we did not observe differences in the rates of major bleeding or mortality between the paroxysmal and persistent AF patients. Moreover, the paroxysmal and persistent AF patients exhibited similar numbers of hospitalizations for cardiovascular or any other reasons. TE events were significantly higher in women, consistent with previous evidence for permanent AF patients [18] but not with previous data for newly diagnosed AF patients [19].

### Antithrombotic treatment

Few data on the correlation of antithrombotic treatment with a given type of AF, AF recurrences and the under- or overtreatment of paroxysmal and persistent AF are available [11,20-22]. In our study, a significantly higher frequency of warfarin use was observed for patients with persistent AF at baseline and at the 6- and 12-month follow-up examinations compared to patients with paroxysmal AF, despite similar CHADS_2_ scores. These data are consistent with previous evidence that nonparoxysmal AF is an independent predictor of warfarin use [21]. Moreover, at the 6- and 12-month follow-up examinations, warfarin was more frequently prescribed for patients with AF recurrences.

The majority of patients underwent cardioversion within 15 days prior to enrollment in the GISSI-AF study. Therefore, if the cardioversion was performed 48 hours after arrhythmia initiation, the patients had an indication for warfarin treatment for at least four weeks according to the guidelines [1,15]. Subsequently, the choice to continue or interrupt warfarin treatment should have been performed after considering the TE risk score of a given patient. This decision was left to the judgment of the referring physician with a strong recommendation to follow the available AF guidelines. However, according to guideline suggestions [15], a significant under- and overtreatment was observed at the 6- and 12-month follow-up examinations, respectively, and was associated with an excess of TE and major bleeding events. In addition to the CHADS_2_ score, persistent AF and AF recurrences were most likely considered to be indications for warfarin treatment by the referring physician.

### Atrial fibrillation recurrence

The time after the spontaneous restoration of SR or cardioversion is a vulnerable period for embolization because rhythm shifts may increase the incidence of TE events. Invasive devices (e.g., a pacemaker or implantable cardioverter-defibrillator memory) have been used to record AF episodes in patients with paroxysmal AF. These devices have demonstrated that an increase in the occurrence, duration, and burden of AF recurrences was associated with an increased frequency of TE events [23-25]. Recently, the ASSERT trial [26] in a large population of patients with no history of AF showed that subclinical atrial arrhythmias (episodes of an atrial rate >190 beats per minute for more than 6 minutes), as detected by implantable devices, were associated with an increased TE risk. The clinical impact of these data remains to be elucidated.

GISSI-AF was the first large trial assessing the influence of non-invasively detected AF recurrences (based on information gathered during scheduled visits and a transtelephonic monitoring device) on TE risk. In our population, the AF recurrence rate was high (51%), one-fourth of which were asymptomatic. The TE event rate was lower, even if not significantly different, in patients without AF recurrences compared to those with both symptomatic and asymptomatic AF recurrences, despite a higher use of warfarin in the latter group.

Our preliminary results need to be confirmed by larger studies. However, our data suggest the utility of non-invasive methods to detect asymptomatic AF recurrences. Even if implantable devices are possibly more efficient in identifying AF recurrences, in our study the high patient compliance with the ECG transtelephonic transmission protocol helped identify a high number of asymptomatic AF recurrences that would otherwise not be recordable.

### Limitations

The present study was a post-hoc analysis of the GISSI-AF trial that aimed to assess an objective different from the evaluation of TE events. The follow-up was limited to 12 months, which may have been too short to evaluate rare complications, such as TE events, major bleeding and mortality. Not all of the AF asymptomatic recurrences were likely to have been detected using our noninvasive method. Finally, the unexpectedly low event rate limited the data interpretation.

## Conclusions

The present cohort of paroxysmal and persistent AF patients showed a very low number of TE and major bleeding events despite the under- and overtreatment with warfarin observed during follow-up. Our results indicate that the TE and major outcome events occurred with a low and similar incidence in patients with paroxysmal or persistent AF. However, even such a low TE event rate may provide information. In a prospective trial, in which the patients with paroxysmal or persistent AF were followed with frequent clinical controls and transtelephonic monitoring, the criteria used by the referring physicians for warfarin administration besides the CHADS_2_ score (particularly the presence of AF recurrences) seemed to be effective for selecting patients who would benefit from anticoagulant therapy.

## Abbreviations

AF: Atrial fibrillation; CHADS2 score: Heart failure, hypertension, age, diabetes, prior stroke or TIA double; GISSI-AF: Gruppo Italiano per lo Studio della Sopravvivenza nell’Infarto Miocardico – Atrial Fibrillation; OBRI score: Age, prior stroke, prior gastrointestinal bleeding, one or more recent myocardial infarctions, hematocrit <30%, serum creatinine concentration >1.5 mg/dl, diabetes; SR: Sinus rhythm; TE: Thromboembolic

## Competing interests

The authors declare that they have no competing interests.

## Authors’ contributions

MD, MGF: concept/design, data analysis, data interpretation, manuscript drafting. SB, CF, GC, GF: data analysis, data interpretation. FC, SQ, LS, LAM: data collection, manuscript revision for important intellectual content. APM, RL: concept/design, manuscript revision for important intellectual content. All of the authors read and approved the final manuscript.

## Pre-publication history

The pre-publication history for this paper can be accessed here:

http://www.biomedcentral.com/1471-2261/13/28/prepub
